# Mechanisms of motivational interviewing in health promotion: a Bayesian mediation analysis

**DOI:** 10.1186/1479-5868-9-69

**Published:** 2012-06-08

**Authors:** Angela G Pirlott, Yasemin Kisbu-Sakarya, Carol A DeFrancesco, Diane L Elliot, David P MacKinnon

**Affiliations:** 1 Department of Psychology, Arizona State University, Tempe, AZ, 85287-1104, USA; 2 Division of Health Promotion and Sports Medicine; Department of Medicine, Oregon Health & Science University, Portland, OR, 97239-3098, USA

**Keywords:** Motivational interviewing, Dietary change, Occupational health, Firefighters, Bayesian mediation

## Abstract

**Background:**

Counselor behaviors that mediate the efficacy of motivational interviewing (MI) are not well understood, especially when applied to health behavior promotion. We hypothesized that client change talk mediates the relationship between counselor variables and subsequent client behavior change.

**Methods:**

Purposeful sampling identified individuals from a prospective randomized worksite trial using an MI intervention to promote firefighters’ healthy diet and regular exercise that increased dietary intake of fruits and vegetables (*n* = 21) or did not increase intake of fruits and vegetables (*n* = 22). MI interactions were coded using the Motivational Interviewing Skill Code (MISC 2.1) to categorize counselor and firefighter verbal utterances. Both Bayesian and frequentist mediation analyses were used to investigate whether client change talk mediated the relationship between counselor skills and behavior change.

**Results:**

Counselors’ global spirit, empathy, and direction and MI-consistent behavioral counts (e.g., reflections, open questions, affirmations, emphasize control) significantly correlated with firefighters’ total client change talk utterances (*r*s = 0.42, 0.40, 0.30, and 0.61, respectively), which correlated significantly with their fruit and vegetable intake increase (*r* = 0.33). Both Bayesian and frequentist mediation analyses demonstrated that findings were consistent with hypotheses, such that total client change talk mediated the relationship between counselor’s skills—MI-consistent behaviors [Bayesian mediated effect: *αβ* = .06 (.03), 95% CI = .02, .12] and MI spirit [Bayesian mediated effect: *αβ* = .06 (.03), 95% CI = .01, .13]—and increased fruit and vegetable consumption.

**Conclusion:**

Motivational interviewing is a resource- and time-intensive intervention, and is currently being applied in many arenas. Previous research has identified the importance of counselor behaviors and client change talk in the treatment of substance use disorders. Our results indicate that similar mechanisms may underlie the effects of MI for dietary change. These results inform MI training and application by identifying those processes critical for MI success in health promotion domains.

## Background

The firefighter stereotype likely elicits an image of a strong hero rescuing individuals from burning buildings. Yet firefighters’ health profiles mirror that of other workers and include an unhealthy diet, a lack of regular physical activity, and being overweight/obese [1]. In addition, occupational hazards increase firefighters’ risk for cancer, heart disease, and musculoskeletal injuries [2-6]. Therefore, firefighters potentially benefit from health promotion programs; however, previous interventions targeting firefighters’ diet and exercise have not been particularly successful [7].

### Overview of PHLAME

The PHLAME (Promoting Healthy Lifestyles: Alternative Models’ Effects) study was a randomized prospective trial of two paradigms to achieve healthy nutrition and regular physical activity. Firefighters from five departments were randomized to one of three conditions: 1) a team-centered peer taught curriculum, which used firefighters’ inherent team structure to promote task cohesion and social norms of healthy diet and exercise; 2) one-on-one counselor-led motivational interviewing (MI) sessions; and 3) a control, assessment-only format. The study’s details, one-year and longitudinal outcomes, and team-centered program’s mediation effects have been reported elsewhere [8-10]. Both interventions achieved moderate effect sizes for improving diet and physical activity [8], and PHLAME is listed on the Cancer Control P.L.A.N.E.T. website for evidence-based programs for both promoting healthy nutrition and enhancing physical activity (http://cancercontrolplanet.cancer.gov/). This project reports findings concerning the mechanism of motivational interviewing’s efficacy in the PHLAME intervention.

### Motivational interviewing (MI)

Motivational Interviewing emphasizes using autonomous motivation described in self-determination theory to elicit change [11,12]. Self-determination theory [13] suggests that two types of motivation exist—autonomous, or motivations to change coming from within the individual, and controlled, or motivations to change coming from outside pressures. Autonomous motivation to change better elicits and sustains change over time than controlled motivation, because the motivation to change is self-driven, rather than driven by the desires of others. In traditional behavior modification counseling, the provider educates the client about his or her unhealthy action(s), the potential adverse consequences and advises the individual to change their behavior. Although intuitively compelling, evidence of adverse effects might seem sufficient to overcome robust defense mechanisms and motivate change, this external motivation model has limited effectiveness [14]. Motivational interviewing (MI) is designed to establish an individual’s autonomous motivation and allows her or him to define a personally relevant change process [11,12]. A counselor using an MI approach elicits, rather than imposes upon, the client’s own reasons for change (autonomous motivation); assists in resolving ambiguity about the decision; collaborates on any change plans; and honors the client’s autonomy. Specific MI skills are well described and include expressing empathy, using accurate reflective listening, asking open-ended questions that elicit reasons to change, rolling with resistance, and affirming the client’s strengths, abilities, and self efficacy [12].

The initial research with MI established its efficacy with addiction and substance abuse [15,16], followed by extending the technique as a means to promote healthy actions [14,17]. More than 200 MI trials have been published and compiled in reviews [18-24]. In recent years, MI has been applied to new behavioral domains (e.g., schizophrenia [25], childhood obesity [26]), and investigators also have examined MI’s inner workings and theoretical structure by establishing the relationship among counselor actions, client comments, and subsequent behavioral outcomes, thus potentially enhancing the efficacy of MI interventions and its application to other domains.

#### *MI & health promotion*

MI has been successful in substance use intervention research [22,27-29], yet its use in health promotion research has not yet been extensively studied [22,30]. The psychological processes underlying cessation of negative behaviors may differ from promotion of positive behaviors; likewise, the psychological processes underlying addictive versus non-addictive behavioral change may differ [31].

For example, using MI for addictive behavior modification, such as smoking or alcohol cessation, requires overcoming psychological *and* physiological resistance, which potentially makes the process more difficult. Feelings of denial, ambivalence, and resistance may be stronger for addictive behaviors than non-addictive behaviors like fruit and vegetable consumption [31]. Indeed, Resnicow et al. [32] found that the major barriers to increasing fruit and vegetable consumption were more pragmatic (e.g., lack of time and availability, taste preference) than psychological or physiological. Thus, the psychology underlying abstinence and relapse may be less relevant to health promotion MI interventions [31]. Finally, the link between cigarette, alcohol, and other substance use and severe health *risks*, such as cancers, are identifiable and accessible, whereas the link between fruit and vegetable consumption and health *benefits* is probably less accessible and seems less certain. The ease of accessibility and severity of possible consequences may enhance one’s motivation to quit. It is therefore important to test MI’s success in health promotion interventions as the strength of the motivation to change might differ and undercut its effectiveness.

#### *MI’s underlying mechanisms: Client change talk*

Client “change talk” (i.e., clients’ utterances of his or her intentions to change) has emerged as a potential intermediate variable between the application of specific MI counseling skills and behavior change [33-35]. The role of change talk is consistent with self-perception theory [36], which suggests that individuals more strongly endorse and may act on attitudes they hear themselves say rather than what others are advising them. Sequential analyses have provided temporal evidence of counselor techniques eliciting client responses more strongly than the reverse causal direction of client responses eliciting counselor techniques [35,37]. However, to date, causal linkage studies only have involved MI use in stopping addictions, primarily with alcohol use [18,35,37-40], along with gambling [41]. This is the first description of MI mediation applied to promoting healthy dietary change. Furthermore, we compared the effects of Bayesian and frequentist mediation analyses. We hypothesize appropriate counselor MI techniques predict increased client change talk, which in turn predicts increased fruit and vegetable intake.

## Methods

PHLAME involvement was voluntary, all information collected was confidential, and participants provided written informed consent. Data was collected in 2002 through 2004, with coding and analyses performed from 2009 through 2011. The Institutional Review Board of the Oregon Health & Science University first approved the study in August of 2000.

### Participant selection

Two hundred and two firefighters participated in the PHLAME motivational interviewing (MI) condition with their MI sessions audiotaped. Participants were assessed at baseline, the MI intervention began soon after, and follow-up measures were obtained one-year after baseline. Purposeful sampling (a technique to obtain a non-representative subset of a larger population to serve a specific purpose) was used to identify approximately 40 individuals for inclusion in these analyses to achieve a range of performance on the outcome variable—daily fruit and vegetable intake—and be feasibly coded for analysis within the study budget. Having a sample in which some participants changed and some did not would hopefully provide sufficient variability on the mediating and outcome variables so as to increase the ability to determine the mechanisms of the MI intervention. Forty three participants’ MI sessions were selected for coding, with 21 indentified as “changers” and 22 indentified as “non-changers.” “Changer” criteria were an increase in fruit and vegetable intake of at least 50%, an initial fruit and vegetable intake of less than 8 servings per day, and weight gain less than 5% of initial body weight. “Non-changer” criteria were an increase or decrease in fruit and vegetable intake of less than 15% and an initial fruit and vegetable intake of less than 8 servings per day. Changers and non-changers were equivalent at baseline on age, years as firefighter, gender, body mass index, general health, intent to eat fruit and vegetables, and autonomous motivation for diet behavior. Table 1 summarizes and compares the baseline assessments of changers, non-changers, and the overall sample.

**Table 1:** Baseline comparison of changers, non-changers and the participant cohort assigned to the Motivational Interviewing intervention

	**Total Motivational Interviewing Cohort**	**Fruit & Vegetable Changers**	**Fruit & Vegetable Non-changers**	***t*-test Comparisons for Changers vs. Non-changers**
	Mean (SD)	Mean (SD)	Mean (SD)	
*n*	202	21	22	*df* = 41
% male	96	100	96	.98
Age	41.1 (8.9)	39.1 (8.4)	42.5 (6.2)	1.54
Years as a firefighter	15.8 (8.7)	14.1 (8.1)	16.4 (8.1)	.92
Servings of fruits and vegetables each day	5.8 (3.4)	3.5 (1.5)	4.9 (1.7)	2.86*
Body Mass Index	27.4 (3.6)	28.4 (4.4)	26.8 (3.0)	−1.42
General Health^#‡^	2.4 (0.8)	2.5 (0.7)	2.4 (0.8)	−.63
Intent to eat fruits and vegetables^#‡^	2.8 (1.2)	2.7 (0.8)	2.6 (1.3)	−.29
Autonomous motivation for diet behavior^#†^	5.6 (1.0)	5.7 (1.0)	5.5 (0.8)	−.57

^#^ Scale items and reliabilities are described in PHLAME addendum at http://www.public.asu.edu/~davidpm/ripl/Phlame.htm..

^‡^ Score based on 5-point scale.

^†^ Score based on 7-point scale and using the TSRQ [42].

* *p* < .01.

### Motivational interviewing intervention

Firefighters met with one of four MI counselors. Prior to the study, each counselor completed approximately 90 hours of MI training, including workshops, educational videotapes, personal coaching from an expert trainer, and practice with standardized patients. Comparable counselor proficiency was established using the Motivational Interviewing Skill Code system (MISC 1.0 [43]), which classifies a counselor’s verbal utterances (statements or single thoughts that end when interrupted or a new statement begins) into mutually exclusive categories. Results were used to calculate indices of MI performance [44]. An independent MISC coder scored each counselor’s practice tapes to verify proficiency. Throughout the study, the counselors met periodically to provide mutual support, debrief challenging interactions, and review MI skills. The structure of their training and resultant fidelity to MI skill criteria have been reported previously [8,45].

Each MI firefighter met at the station with his or her counselor for a series of at least four 30–60 minute one-on-one sessions. The first meeting discussed study timeline and goals and client’s health and values; the second meeting included a review of the firefighter’s baseline testing results (e.g., dietary indices, fitness measures, body weight); and the third and fourth sessions continued to focus on achieving the clients’ objectives relative to the study goals of increasing daily servings of fruits and vegetables each day, enhancing daily physical activity, and achieving a healthy body weight. The second MI session was selected for coding to capture the client’s reaction to his or her test results and potential client change talk in response to those results.

### Coders and coding

Two research assistants at Oregon Health & Sciences University were trained to use the Motivational Interviewing Skills Coding System (MISC 2.1) in approximately 80 hours over eight weeks. The MISC instrument was developed and refined as a method for evaluating the content of motivational interviewing. The MISC manual, including its rationale, development, and explicit instructions, is available at http://casaa.unm.edu/download/misc.pdf[44]. The MISC has 15 mutually exclusive utterance categories for the counselor’s speech, four categories for client speech, and six “global” scores for the overall interaction.

Once familiar with the coding categories, the research assistants proceeded through a series of learning tasks using fictional transcripts. The tasks included: 1) identifying counselor utterances categories; 2) categorizing client change talk into specific categories; and 3) assigning code global scores (evocation, collaboration, autonomy support, direction, self-exploration).

For accurate coding, the audiotapes were transcribed. Following training, the 43 selected participants’ tapes and transcripts were randomly assigned to the coders, who were unaware of the participant’s identity and outcomes. Coders then both listened to and reviewed the transcripts using a modified MISC coding system. Coding an interaction took three times the length of a recorded session, thus a 45-minute session required more than two hours to code.

For the counselor behaviors, utterance codes for the 15 major categories were tallied and MI-consistent and MI-inconsistent behaviors were summed for each interaction. *MI-consistent behaviors* were the combined counts of the following utterance categories: affirm, advise with permission, emphasize control, ask open question, reflect, reframe, and support. *MI-inconsistent behaviors* consisted of summing the following utterance categories: confront, advise without permission, direct, raise concern without permission, and warn [44].

Coders also rated the overall interaction on the following five counselor global categories: *evocation* (counselor elicits client change talk)*, collaboration* (counselor supports and explores the client’s own concerns)*, autonomy-support* (counselor emphasizes the client’s freedom of choice)*, empathy* (counselor is interested in client’s perceptions, situations, and feelings), and *direction* (counselor directs client to targeted behaviors) on a 5-point scale, whereby 1 indicated the lowest degree of that construct and 5 indicated the highest level. An *MI spirit* construct was calculated based on averaging the collaboration, evocation, and autonomy-support items [44].

Firefighters’ utterances were coded based on MISC client change talk categories and identified as *total positive client change talk* and *total client sustain talk*, which indicated intentions to change behaviors and sustain the status quo, respectively. The mutually exclusive categories included the positive and negative valences of the categories of client commitment language, client taking steps, and client change talk. *Total positive client change talk* was the sum of positive client change talk, positive client commitment language, and positive client taking steps, and *total client sustain talk* was the sum of sustain client change talk, sustain client commitment language, and sustain client taking steps. Coders also provided an overall global rating (on a 7-point scale, 7 indicating the highest level) of the clients’ engagement and self-discovery, called *self-exploration*.

The outcome behavior was dietary change in fruit and vegetable intake, assessed by number of servings of fruits and vegetables per day, using a previously validated self-report instrument [46]. The change score was computed by subtracting the baseline daily fruit/vegetable intake score from the one-year post-treatment fruit/vegetable intake score, with higher scores indicating greater increase in fruit and vegetable intake.

### Inter-rater reliability

Ten percent of sessions (4 interactions) were randomly selected and coded by both research assistants to assess inter-rater reliability. Transcripts rated by both coders were matched utterance by utterance. Utterance codes forming MI-consistent and MI-inconsistent counselor behaviors were combined separately for ease of interpretation. The cross-tabulated frequencies of counselor and client utterance counts given in Table 2 indicate a high agreement between coders. The contingency coefficient—a nonparametric equivalent of the correlation coefficient—was calculated for reliability since Cohen’s kappa was undefined due to the nature of the data (i.e., not every category existed for both set of coders). The overall contingency coefficient was *C* = 0.91, suggesting excellent inter-rater reliability [47]. Table 2 reports each rater’s frequency codes to reflect the inter-rater reliability.

**Table 2:** Cross-tabulated frequencies of behavior counts rated by each coder for 4 interactions (approximately 10% of total)

				**Coder 1**									
				**Counselor Utterances**		**Client Utterances**							
						**Total Positive Client Change Talk**			**Total Sustain Talk**				
				**MICO**	**MIIN**	**Positive Client Change Talk**	**Positive Client Commitment Language**	**Positive Client Taking Steps**	**Negative Client Change Talk**	**Negative Client Commitment Language**	**Negative Client Taking Steps**	**Other**	**Total**
**Coder 2**	**Counselor Utterances**		**MICO**	174	0	0	0	0	0	0	0	7	181
			**MIIN**	0	0	0	0	0	0	0	0	0	0
	**Client Utterances**	**Total Positive Client Change Talk**	**Positive Client Change Talk**	0	0	93	0	0	1	0	0	3	97
			**Positive Client Commitment Language**	0	0	0	4	0	0	1	0	0	5
			**Positive Client Taking Steps**	0	0	1	0	11	0	0	0	0	12
		**Total Client Sustain Talk**	**Negative Client Change Talk**	0	0	0	0	0	17	0	0	0	17
			**Other**	11	0	6	0	0	0	0	0	493	510
			**Total**	185	0	100	4	11	18	1	0	503	822

*Note.* MICO = MI-consistent counselor behaviors; MIIN = MI-inconsistent counselor behaviors. Coder 1 did not rate any negative commitment language or taking steps behaviors. Other = all other counselor and client behaviors (i.e., giving information, filler, facilitate, closed question, neutral, structure).

## Results

### Participants

Descriptive findings for the changer and non-changer participants, along with the entire MI cohort, are presented in Table 1. For the participants whose data was coded (*n* = 43), participants were mainly male (98%) and white (non-Hispanic, 95%), with a mean age of 41 (*SD* = 7.46) at the start of the study. At baseline year, the average servings of fruits and vegetables per day were 4.29 (*SD* = 1.76) and participants’ mean body mass index was 27.6 (*SD* = 3.8).

### Descriptive and correlational results

Table 3 presents the descriptive statistics of each variable and Table 4 presents the correlations between the client and counselor utterances and fruit/vegetable intake. Consistent with predictions, counselor spirit, empathy, direction and MI-consistent behaviors correlated positively with positive total client change talk (*r*s = .42, .40, .30, and .61, respectively). In addition, counselor spirit, empathy, and direction correlated positively with client self-exploration (*r*s = .61, .66, and .48, respectively). Total positive client change talk and self-exploration correlated positively with fruit/vegetable intake (*r*s = .33 and .28, respectively). Counselor MI-inconsistent skills did not correlate with positive client change talk or self-exploration, but marginally correlated with fruit/vegetable intake (*r* = .28).

**Table 3:** Descriptive Results

**Variable**			**Mean (SD)**	**Minimum**	**Maximum**
**Counselor Variables**					
	*MI-Consistent Behaviors*		51.81 (23.18)	16.00	101.00
		Affirm	3.70 (2.87)	0.00	10.00
		Advise with Permission	0.34 (.92)	0.00	5.00
		Emphasize Control	4.07 (2.85)	1.00	13.00
		Ask Open Question	8.67 (6.20)	0.00	26.00
		Reflect	33.28 (16.58)	7.00	69.00
		Reframe	0.14 (0.35)	0.00	1.00
		Support	1.63 (2.44)	0.00	13.00
	*MI-Inconsistent Behaviors*		0.60 (1.45)	0.00	7.00
		Confront	0.12 (.39)	0.00	2.00
		Advise without Permission	0.35 (1.09)	0.00	5.00
		Direct	0.12 (0.39)	0.00	2.00
		Raise Concern without Permission	0.02 (0.15)	0.00	1.00
		Warn	0.00 (0.00)	0.00	0.00
	*Global Counselor Scores*				
		MI Spirit (Collaboration, Autonomy, Evocation)	11.11 (1.55)	6.00	14.00
		Evocation	3.65 (0.75)	1.00	5.00
		Collaboration	3.35 (0.70)	2.00	5.00
		Autonomy-Support	3.93 (0.34)	3.00	5.00
		Empathy	3.67 (0.78)	2.00	5.00
		Direction	4.26 (0.85)	2.00	5.00
**Client Variables**					
	*Total Positive Client Change Talk*		25.95 (13.41)	1.00	60.00
		Positive Client Change Talk	21.58 (11.81)	1.00	60.00
		Positive Client Commitment Language	0.84 (1.51)	0.00	6.00
		Positive Client Taking Steps	3.02 (2.96)	0.00	12.00
	*Total Client Sustain Talk*		4.35 (4.84)	0.00	26.00
		Sustain Client Change Talk	4.14 (4.13)	0.00	19.00
		Sustain Client Commitment Language	0.19 (1.07)	0.00	7.00
		Sustain Client Taking Steps	0.02 (0.15)	0.00	1.00
	*Global Client Scores*				
		Self-Exploration	4.30 (1.32)	2.00	7.00
Client Outcome Variable					
	*Fruit & Vegetable Consumption*				
		Fruit & Vegetable Consumption (Baseline)	4.20 (1.76)	1.34	7.85
		Fruit & Vegetable Consumption (Follow-Up)	6.21 (4.11)	0.23	24.84
		Fruit & Vegetable Change Score	1.81 (4.36)	−3.87	23.50

**Table 4:** Correlations

**Variable**	**2.**	**3.**		**4.**		**5.**		**6.**		**7.**	**8.**		**9.**	
*Counselor utterances*														
Behavior counts														
1. MI-Consistent	−0.02	0.38	**	0.48	**	0.19		0.61	***	0.23	0.23		0.05	
2. MI-Inconsistent		−0.09		−0.12		0.16		−0.04		0.03	−0.06		0.28	+
Global scores														
3. Spirit (Collaboration, Autonomy, Evocation)				0.55	***	0.45	**	0.42	**	0.16	0.61	***	0.14	
4. Empathy						0.49	**	0.40	**	0.11	0.66	***	0.19	
5. Direction								0.30	+	0.13	0.48	**	0.05	
*Client utterances*														
Behavior counts														
6. Total Positive Client Change Talk										0.23	0.46	**	0.33	*
7. Total Client Sustain Talk											0.26	+	−0.10	
Global scores														
8. Self-exploration													0.28	+
*Client Outcome Variable*														
9. Fruit/Vegetable Change Score														

***p < .001, **p < .01, *p < .05, + *p* < .10.

Given counselor MI-inconsistent behaviors did not correlate with our hypothesized mediator, we did not perform mediation analyses using MI-inconsistent counselor behaviors. Client sustain talk did not correlate significantly with any counselor scores or fruit and vegetable consumption; we therefore dropped this variable from subsequent analyses.

### Mediation analyses

**Figure 1:**
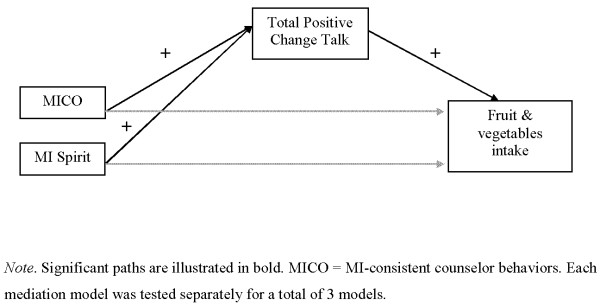
**Mediation models tested.***Note*. Significant paths are illustrated in bold. MICO = MI-consistent counselor behaviors. Each mediation model was tested separately for a total of 2 models.

Mediation analyses help to understand the underlying mechanisms of a phenomenon [48]. For example, a simple mediated effect occurs when an intervention changes a mediator (i.e., the *α* path) and that mediator changes the outcome (i.e., the *β* path) (see Figure 1). The mediated effect is then the product of *α* and *β* paths, *αβ*, which estimates the part of the total program effect transmitted through the mediator. The analysis of mediated effects in prevention programs can be sometimes problematic if the sample size is small. In that case, the sampling distribution of the mediated effect estimate can have a non-normal distribution, which leads to biased confidence interval estimates. An approach to this problem is to use Bayesian analyses to estimate the mediated effect, since a Bayesian approach does not require the assumption of normality in the sampling distribution of estimates [49]. Another advantage of the Bayesian approach is that it allows the statistical incorporation of prior findings into the statistical analysis. In Bayesian analyses, the unknown parameters are treated as random variables with a distribution. The mediated effect estimator is updated by using the prior knowledge of estimators which are assumed to have a probability distribution called the prior distribution. We used MPlus 6.0 software to conduct the Bayesian mediation analyses.

Given our significant correlational findings and the previous literature suggesting a mediational relationship between counselor behaviors (MI-consistent behaviors and spirit) and hypothesized mediator (total positive client change talk), and with the mediator and the outcome variable (fruit and vegetable consumption), we performed a mediation analysis examining whether total positive client change talk mediated the relationship between counselor behaviors (MI-consistent behaviors and spirit) and fruit/vegetable consumption (See Figure 1). Due to the limited sample size, we analyzed each hypothesized mediated effect separately. To clarify, these mediation analyses were not examining the mediators of the overall intervention (i.g., MI intervention versus control) but instead mediators of the efficacy of the MI intervention on fruit-vegetable consumption.

#### *Bayesian mediation analysis*

Bayesian mediation analyses allow for the incorporation of prior research. Apodaca & Longabaugh’s [50] meta-analysis indicated that the pooled effect size for MI counselor behavior and client behavior was *r* = .46, and that the pooled effect size for client behavior and outcome variables was *r* = .23. In a mediated effect, correlation coefficients are suggested as appropriate effect size estimates of individual paths [51]. Consequently, we used the above correlation coefficients as informative priors in the Bayesian mediation analyses (MPLUS syntax provided in Appendix A).

First, we performed a Bayesian mediation analysis with total positive client change talk mediating the relationship between MI-consistent counselor behaviors and fruit/vegetable consumption. The posterior mean of the mediated effect of counselor MI-consistent behaviors through total positive client change talk on change in fruit/vegetable consumption was *αβ* = .06 (.03) with a 95% credible interval [.02, .12]. This supports the hypothesis that the effect of counselor MI-consistent behavior on the outcome variable is mediated through total positive client change talk. MI-consistent counselor behaviors predicted an increase in total positive client change talk, and in turn, total positive client change talk increas ed fruit and vegetable consumption.

Next, we performed a Bayesian mediation analysis of total positive client change talk mediating the relationship between counselor spirit and fruit/vegetable intake. A similar pattern was observed for counselor spirit: the posterior mean of the mediated effect of counselor spirit through total positive client change talk on change in fruit and vegetable consumption was *αβ* = .06 (.03) with a 95% credible interval [.01, .13]. Counselor spirit increased total positive client change talk, and client total positive client change talk increased fruit and vegetable consumption.

The significant mediation paths for both sets of Bayesian analyses are illustrated in Figure 1, with Table 5 listing the path estimates for the mediation analyses.

**Table 5:** Path estimates (posterior standard deviation) for single mediator models using Bayesian mediation

	**Outcome Variables**	
Predictor Variables	Total Positive Client Change Talk (M)	Change in Fruit & Vegetable Consumption (Y)
	β (*SE*)	β (*SE*)
*Model 1*		
MI-Consistent (X)	0.38** (0.07)	−0.05 (0.04)
Total Positive Client Change Talk (M)		0.16** (0.06)
*Model 2*				
MI Spirit (X)	0.49** (0.14)	−0.11 (0.52)		
Total Positive Client Change Talk (M)		0.12* (0.06)		

** *p* < .01, * *p* < .05.

#### *Frequentist (non-Bayesian) mediation analysis*

We also conducted conventional frequentist mediation analyses to compare the estimates from the two approaches, using PRODCLIN [52] to calculate the 95% asymmetric confidence limits for each mediated effect. The PRODCLIN program provides more accurate confidence limits for the mediated effect since the distribution of the product of two normally distributed variables is not normal [52].

The mediated effect of counselor MI-consistent behaviors through total positive client change talk on change in fruit and vegetable consumption was significant: *αβ* = .05 (.02) with a 95% confidence interval of [.01, .10], as was the mediated effect of counselor’s MI spirit through total positive client change talk on change in fruit and vegetable consumption: *αβ* = .37 (.23) with a 95% confidence interval of [.003, .89], both consistent with the Bayesian mediation analyses. These results suggest that total positive client change talk mediates the relationship between MI-consistent counselor behaviors and spirit and the outcome variable—increased fruit and vegetable consumption.

The standard errors are slightly smaller and confidence intervals narrower when using the Bayesian approach compared with the frequentist approach. However, the meaning of the interval differs by method. A 95% Bayesian credible interval suggests that there is a 95% chance that the credible interval contains the true value of the coefficient based on the observed data, whereas a 95% frequentist confidence interval means that if we repeatedly sample from a population and calculated the confidence interval for each sample, on average 95% of those intervals contain the true value of the estimate. In this sense, Bayesian credible intervals are more intuitive than conventional confidence intervals, since credible intervals rely on a more meaningful probability distribution rather than an idealistic assumption of repeated sampling under identical conditions [49].

## Discussion

Overall, our findings are that counselor behavior (MI-consistent behaviors and spirit) predicts firefighters’ expressions of intentions to make positive changes, and those expressions in turn predict increased future fruit and vegetable intake. This parallels findings with the use of MI for stopping harmful health behaviors (e.g., alcohol use and smoking) and also supports both components of the proposed theoretical structure of MI [34]. MI spirit has been suggested as a critical feature in its efficacy [53], as it relates to the tenant that an empathic relationship displaying positive regard can result in favorable outcomes [54-56]. Previously, the mediational link between spirit and client behavior change has been found for alcohol [37] and smoking [57]. However, those are both situations where the guilt associated with the action being addressed might heighten the adversarial role of counselor and client, augmenting the importance of positive regard. Finding a relationship with spirit when addressing fruit and vegetable intake highlights the importance of collaboration and honoring autonomy.

For counselor behaviors and overall spirit, total positive client change talk was the mediating variable, and has become an important construct in MI. Proposed as an instrumental factor in 2002 [58], its role has been supported by the work of investigators using sequential analysis of MI interactions with alcohol use [35,38]. Our findings corroborate those findings and extend its relevance to promoting healthy behaviors. Client change talk’s role also supports MI’s proposed underpinnings in self-perception theory [36,59] that suggest the counterintuitive sequence of an individual’s behaviors affect their attitudes, as in this case they may talk themselves into changing. Finding that total positive client change talk mediated behavioral outcomes underscores the importance of MI counselors’ use of techniques to facilitate those expressions.

Moyers et al. [35] tested a directional model of motivational interviewing efficacy for an alcohol cessation intervention, whereby MI-consistent counselor behaviors elicited participants’ client change talk 17% of the time and suppressed participants’ sustain talk. This provides specific directional evidence suggesting counselor behaviors evoke client responses, rather than client responses eliciting counselor behaviors or a bidirectional model of causality. Using this framework, we tested whether counselor behaviors elicited change in total positive client change talk which in turn predicted change in fruit and vegetable consumption. Our findings are consistent with total positive client change talk as a mediator of the relationship between counselor behaviors and fruit and vegetable intake. Although we did not use sequential analyses of counselor and client utterances (which provide temporal evidence of the causal relationship between predictor and mediator variables), our outcome variable was assessed at a second time point. This provides some temporal evidence for client and counselor behaviors eliciting changes in fruit and vegetable consumption.

MI has been successful in substance use intervention research [22,27-29] yet its use in health promotion research has not yet been extensively studied [30]. Psychological factors underlying cessation interventions potentially enhance the importance of the intervention and thus augment its efficacy. It is therefore important to test MI’s success in health promotion interventions as the strength of the motivation to change might differ and undercut the effectiveness. Our results confirm relationships previously identified with stopping harmful behaviors also apply to MI use with promoting healthy actions.

An interesting and perhaps counterintuitive finding was the positive correlation between MI-inconsistent behaviors and fruit and vegetable consumption. However, MI-inconsistent behaviors occurred infrequently, included giving advice or directing without permission, rather than warning or confronting (see Table 3), and did not correlate with total positive client change talk. One could speculate that unsolicited advice on increasing fruit and vegetable intake may be helpful for increasing the behavior but because of the low frequency it is important to not overstate this finding. That MI-inconsistent behaviors were unrelated to client positive change talk suggests MI-inconsistent behaviors did not elicit spontaneous client utterances of intentions to change behaviors, but still increased fruit and vegetable consumption. An interesting future direction might be to examine the relationship between MI-inconsistent counselor behaviors and long-standing changes.

### Limitations & future directions

Our participants were mainly white males, which may limit the generalization of our findings; however, efficacy trials indicate that MI effect sizes may be greater in minority populations [20,60]. In addition, our sample size was relatively small, which can hinder tests of mediation particularly because the mediated effect estimate has a non-normal distribution. Furthermore, we used purposeful sampling to identify a subset of participants for which a range of change occurred on the outcome variable, and so therefore it is also possible that confounders may have affected the mediation analysis. To overcome our limited sample size, we used a novel approach—Bayesian mediation analyses (which does not require normal distributions of the estimates [49])—to investigate mediation. Bayesian mediation analyses supplements traditional mediation analyses in small sample sizes by using prior information on the parameter as well as making fewer assumptions about the distribution of the mediated effect. Even given our sample, total positive client change talk emerged as a significant mediator between counselor behaviors and fruit and vegetable consumption, thus suggesting the robustness of the results.

An additional limitation is that our coding data came from a single interaction between the counselor and the firefighter. Recent work on client change talk has focused on the trajectory of change within and across sessions [41,61], and this trajectory has been found to be important, with differences emerging over time [33]. However, identifying mediation effects from the initial interaction in which clients discussed their assessment demonstrates the robustness of the findings. Perhaps the conversation following the discussion of the firefighters’ assessment presents the strongest opportunity for eliciting client change talk—change talk that has the strongest and lasting impact on health behaviors. Future research can compare the differential impact of the motivational interviewing immediately after the results assessment (session two) with the other follow-up sessions to determine the critical sessions for behavior change in the motivational interviewing intervention.

## Conclusion

Motivational interviewing as an intervention is a resource- and time-intensive process, and it is currently being applied in many arenas. Therefore it is imperative to understand the mechanisms of motivational interviewing’s effectiveness to inform MI training and application, ideally identifying those resource and time intensive processes critical for MI success in health promotion domains.

## Appendix A

MPlus syntax for the Bayesian mediation analysis

### DATA

File = C:\Documents and Settings\MIdata.dat;

### VARIABLE

Missing = all (999); Names = ID MICO Spirit changetalk FVdiff; Usevariables = MICO changetalk FVdiff;

### MODEL

! Estimating and labeling α and β parameters for the mediation model; changetalk on MICO (a);FVdiff on changetalk (b); MICO;

### ANALYSIS

! Bayesian estimation (Markov Chain Monte Carlo Algorithm);Estimator = BAYES;! Specifying the mean and standard error of informative priors;

### Model Priors

a ~ N(0.46, 0.019);b ~ N(0.23, 0.022);

### Model Constraint

new (indirect);indirect = a*b;

### OUTPUT

! Tech8 gives scale reduction factor;tech1 tech8 standardized;

### PLOT

! Plot2 gives the times series (trace) plots for each parameter;type = plot2;

## Competing interests

The PHLAME program is listed on the Cancer Control P.L.A.N.E.T. website of evidence-based programs, and the PHLAME team program is distributed through the Center for Health Promotion Research at Oregon Health & Science University (OHSU). OHSU and Dr. Elliot have a financial interest from the commercial sale of technologies used in this research. This potential conflict of interest has been reviewed and managed by the OHSU Conflict of Interest in Research Committee.

## Authors’ contributions

DE and CAD conducted the original study; YK-S and DPM performed the statistical analyses; and AGP, YK-S, and DPM drafted the manuscript. All authors contributed to and approved the final manuscript.
